# Uncovering the impact of infection routes on within-host MPXV dynamics: Insights from a mathematical modeling study

**DOI:** 10.1371/journal.pcbi.1013073

**Published:** 2025-05-19

**Authors:** Qi Deng, Woldegebriel Assefa Woldegerima, Wenjing Zhang, Ali Asgary, Jude Dzevela Kong, Sarah Flicker, Nicholas H. Ogden, James Orbinski, Nicola Luigi Bragazzi, Jianhong Wu

**Affiliations:** 1 Department of Mathematics and Statistics, York University, Toronto, Ontario, Canada; 2 Department of Mathematics and Statistics, Texas Tech University, Lubbock, Texas, United States of America; 3 Global South Artificial Intelligence for Pandemic and Epidemic Preparedness and Response Network, York University, Toronto, Ontario, Canada; 4 Artificial Intelligence and Mathematical Modelling Lab, University of Toronto, Toronto, Ontario, Canada; 5 Department Environmental Studies, York University, Toronto, Ontario, Canada; 6 National Microbiology Laboratory, Public Health Agency of Canada, Saint-Hyacinthe, Québec, Canada; 7 The Dahdaleh Institute for Global Health Research, York University, Toronto, Ontario, Canada; 8 Department of Food and Drugs, University of Parma, Parma, Italy; Pennsylvania State University, UNITED STATES OF AMERICA

## Abstract

The unprecedented mpox outbreak in non-endemic regions during 2022–2023, which has seen a recent resurgence in late 2023–2024, poses a significant public health threat. Despite its global spread, the viral dynamics of mpox infection and the specific characteristics driving these outbreaks remain insufficiently explored. We develop mathematical models to examine the interactions between host immune responses and the virus across three distinct infection routes (intravenous, intradermal, and intrarectal). The models are calibrated using viral load data from macaques infected through each of these three infection routes. Subsequently, we calculate the infectiousness of each infected macaque, finding that the proportion of presymptomatic infectiousness is highest in those infected via sexual contact, followed by skin-to-skin contact. These observations demonstrate that close contact during sexual activity is a significant route of viral transmission, with presymptomatic spread playing a crucial role in the 2022–2023 multi-country outbreak and potentially also in the 2023–2024 multi-source outbreak. Leveraging model predictions and infectiousness data, we assess the impact of antiviral drugs on interventions against mpox infection. Model simulations suggest that early administration of antiviral drugs can reduce peak viral loads, even in individuals with compromised immunity, particularly in cases of infection through skin-to-skin and sexual contact. These results underscore the importance of initiating antiviral treatment as early as possible for mpox-infected patients with compromised immune systems, such as those who are HIV-positive.

## Author summary

We combine mathematical models and experimental data to analyze the within-host mpox infection dynamics and identify the features driving to the 2023-2024 outbreak. Our findings reveal that close contact during sexual activity is a significant route of viral transmission, with presymptomatic spread playing a crucial role in the 2022-2023 multi-country outbreak and potentially also in the 2023-2024 multi-source outbreak. Further, we evaluate the impact of antiviral drugs on interventions against mpox infection. Model simulations suggest that early administration of antiviral drugs can effectively reduce peak viral loads, even in individuals with compromised immunity.

## Introduction

The mpox virus (MPXV), the causative agent of mpox (formerly known as monkeypox), belongs to the *Orthopoxvirus* genus within the *Poxviridae* family [1, 2]. This family also includes the virus responsible for smallpox. MPXV was first discovered in 1958 from pustular lesions in monkeys bred for research [3]. Since the identification of the first human case in 1970 in the Democratic Republic of the Congo, mpox infections have primarily circulated in West and Central Africa [4]. Cases outside these regions were historically rare and typically associated with travel to endemic areas or contact with rodents imported from those regions. However, in May 2022, a significant outbreak occurred outside Africa, with 110 countries reporting approximately 87,000 cases and 112 deaths [5]. Unlike previous transmission patterns, this outbreak primarily affected men who have sex with men (MSM) [6]. The dramatic increase in global mpox cases since 2022 prompted the World Health Organization (WHO) to declare mpox a Public Health Emergency of International Concern (PHEIC) twice within these two years [5].

**Fig 1:**
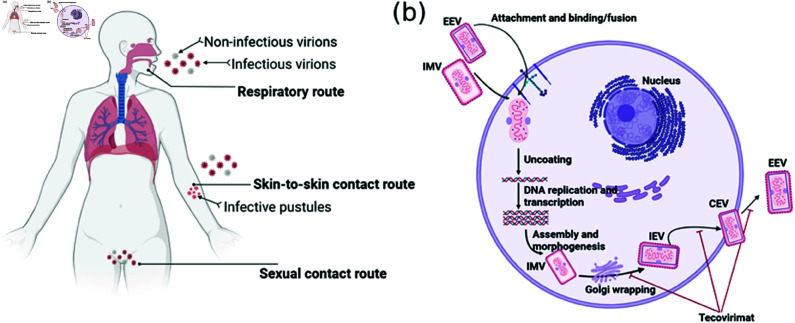
Schematic of MPXV invasion of the host. (a) Three infection routes of mpox: respiratory route, skin-to-skin contact route and sexual contact route. During each contact, the infected individual sheds both infectious and non-infectious virions. (b) A schematic of MPXV infecting the host cell. Both EEV and IMV can invade the cell through unknown receptors or extracellular matrix components. Following viral attachment, the basic steps for mpox life cycle includes uncoating of viral core, viral DNA replication and translation, virus assembly and morphogenesis, and virus release. Figures generated with BioRender (https://biorender.com/).

MPXV is a double-stranded DNA virus capable of entering the host through various routes, including respiratory droplets, skin-to-skin contact, and sexual transmission [2, 7] (Fig 1A). However, the precise target cells for MPXV after entry remain unclear. It has been suggested that several types of mammalian cells, such as monocytes, can be infected by MPXV, as poxviruses are known to attach to and enter host cells without the need for specific receptors [8]. Fig 1B outlines the basic steps of MPXV infecting a target cell. There are two distinct forms of infectious virions: intracellular mature virion (IMV) and extracellular enveloped virion (EEV) [9]. The IMV, which has a single outer lipoprotein bilayer enclosing the viral core, infects target cells via macropinocytosis, while the EEV, which has an additional membrane compared to IMV, infects target cells through fusion with the plasma membrane [10, 11]. As illustrated in Fig 1B, gene replication and transcription occur in the cytoplasm of the target cell, also referred to as the viral factory. After translation, viral proteins are assembled into IMVs, which are further wrapped by Golgi membranes to form intracellular enveloped virions (IEVs). IEVs then fuse with the target cell membrane, leading to the formation of cell-associated enveloped virions (CEVs), which are subsequently released as EEVs [11]. Notably, IMVs are primarily responsible for viral infection within the target cell, while EEVs facilitate viral transmission between cells [12]. Both IMVs and EEVs are released during the lysis of the infected cell.

Common symptoms of mpox infection include headache, fever, and skin lesions, with lymphadenopathy being a distinguishing feature that sets mpox apart from other poxvirus infections, such as smallpox [13]. Currently, there are no antiviral drugs specifically approved for mpox infection. However, antiviral drugs approved for smallpox treatment may be effective in managing severe mpox infections [14]. For example, tecovirimat, which can be prescribed for severe mpox cases, works by inhibiting the function of an essential envelope protein required for EEV production [15, 16] (Fig 1B). Most patients recover fully within 2 to 4 weeks without medical intervention [17], indicating that the immune system plays a crucial role in controlling MPXV replication. As with other viral infections, the host immune system is expected to activate various defense mechanisms following MPXV invasion. The innate immune response, the first line of defense against viral infection, involves the activation of macrophages, dendritic cells, and natural killer cells that recognize and attack MPXV. Subsequently, the adaptive immune response, particularly T cells and B cells, is activated to combat mpox infection by producing cytotoxic responses and specific antibodies that kill infected cells and neutralize the virus [18, 19]. However, the specific mechanisms of host immunity against mpox infection are not yet fully understood, especially in the context of the unprecedented MSM transmission route observed during the 2022 outbreak.

Mathematical models have been extensively used to understand the pathogenesis of various pathogens, including HIV, HPV, influenza, and SARS-CoV-2 [20–30]. These models also provide valuable insights into the transmission dynamics of mpox at the population level [31, 32]. However, the within-host dynamics of mpox infection have not been thoroughly investigated, which could be crucial for the development of anti-MPXV drugs and vaccine therapies. In this paper, we propose mathematical models of ordinary differential equations (ODEs) to study the within-host dynamics of mpox infection by examining the interaction between the host immune response and the virus. The models are fitted to macaque viral load data collected from three distinct infection routes. Then by using the best-fit model, we calculate the infectiousness of individuals following mpox infection to understand the predominance of mpox infections in the MSM community. Further, we gain insights into virus dynamics and immunity in the context of the current outbreak and further test potential therapies through numerical simulations.

## Materials and methods

### Experimental data

Macaque models are the most commonly used animal models to study the pathogenesis of viral infections and to test the efficacy of various potential drugs or vaccines [33, 34]. Macaques infected with MPXV exhibit symptoms and characteristics similar to those observed in humans, indicating that these models are suitable for studying the in vivo interactions between host cells and MPXV, which cannot be explicitly studied in humans.

In an experimental study by Aid and colleagues [35], 18 adult macaques were inoculated with MPXV (lineage B.1, clade 2b; BEI NR-58622) via three different routes of infection: intravenous (IV), intradermal (ID), and intrarectal (IR). Each route mimics the modes of infection in humans under specific circumstances. The IV route involves the direct introduction of pathogens into the bloodstream, modeling diseases transmitted through direct blood contact. The ID route simulates disease transmission through skin-to-skin contact, while the IR route models infections that occur through sexual contact. Of the 18 macaques, 12 were infected with MPXV via the IV route, divided into three groups of four, each receiving doses of 10^6^ TCID50 (50% tissue culture infective dose), 10^5^ TCID50, and 10^4^ TCID50, respectively. The remaining six macaques were evenly divided into two groups, each inoculated with a dose of 10^6^ TCID50 of MPXV via the ID and IR routes, respectively. Plasma viral DNA levels were assessed on days 0, 3, 7, 10, 14, 21, and 28 following infection [35]. The values peaked on day 10 following IV infection and on day 7 following ID and IR infections. Given that the current mpox outbreak primarily spreads through skin-to-skin and sexual contact, especially among MSM, the ID and IR routes effectively model this outbreak [35]. Therefore, these data sets can be combined with our mathematical models to deepen our understanding of mpox pathogenesis and its characteristics in the context of the current outbreak.

### A basic MPXV dynamic model

The exact target of MPXV infection remains unclear. Multiple studies have demonstrated that MPXV effectively infects and replicates within diverse cell types, including epithelial cells and antigen-presenting immune cells [2]. In this study, we focus on infections in monocytes, due to the following two reasons. Firstly, monocytes are identified as primary targets in the orthopoxvirus infection pathway, being rapidly recruited to the site of infection [36, 37]. Further, the presence of replicating smallpox virus (instead of phagocytosed antigen) in monocytes was confirmed in an experiment [38]. Given the high degree of similarity in the DNA sequences among orthopoxviruses, we hypothesize that MPXV predominantly targets monocytes upon entry into the body. Secondly, monocytes circulate in the bloodstream and are able to migrate into tissues, making it a potential vehicle for MPXV to spread from the initial infection site to other organs [2]. Therefore, studying infections in monocytes facilitates a deeper understanding of the initial interactions between MPXV and the host immune response, which are critical for determining the trajectory of the infection.

We first develop a basic within-host model that incorporates only the susceptible monocytes (*T*), infected monocytes in the eclipse phase (the stage between viral attachment and the production of new virus, *I*_1_), productively infected monocytes (*I*_2_), and the MPXV measured in plasma (*V*), which may contains both infectious and non-infectious viruses. Then the interaction between monocytes and MPXV is described by the following ODEs:


$$\left\{ \begin{array}{c} \frac{dT}{dt}=\lambda_{1}-d_{1}T-\beta TV,\\ \frac{dI_{1}}{dt}=\beta TV-kI_{1},\\ \frac{dI_{2}}{dt}=kI_{1}-\delta I_{2},\\ \frac{dV}{dt}=pI_{2}-c_{0}V. \end{array}\right.$$ (1)

In model (1), susceptible monocytes are recruited with a constant rate $\lambda_{1}$, die at a rate *d*_1_, and are assumed to be infected by MPXV to enter the eclipse stage with rate constant β. Infected monocytes in the eclipse stage progress to being productively infected at a rate *k*. Productively infected cells die with a constant rate δ. The parameter *p* is the viral production rate per productively infected cell. *c*_0_ is the base viral clearance rate. All parameters in the model (1) are assumed to be positive. In fact, model (1) represents a basic viral dynamics widely used to study within-host dynamics of many viral infections, however, the target cells in this model differ from those in other studies.

### Models with immune responses

We extend the basic model (1) by considering immune responses. Following viral infections, the host’s innate immune response serves as the first line of defense. Johnston *et al*. [39] found that interferon (IFN) can substantially inhibit mpox infection, and Aid *et al*. [35] also observed an upregulation of IFN in all infected macaques on day 1 following challenge. Therefore, we include the IFN (*F*) into the model to represent the innate immune response against mpox infection. IFNs are produced from infected cells and bind to the receptors on the neighboring target cells, making them less susceptible to infection [40]. Thus the first extended model with innate immune response is:


$$\left\{ \begin{array}{c} \frac{dT}{dt}=\lambda_{1}-d_{1}T-\frac{\beta }{1+\Gamma F}TV,\\ \frac{dI_{1}}{dt}=\frac{\beta }{1+\Gamma F}TV-kI_{1},\\ \frac{dI_{2}}{dt}=kI_{1}-\delta I_{2},\\ \frac{dV}{dt}=pI_{2}-c_{0}V,\\ \frac{dF}{dt}=sI_{2}-\rho F, \end{array}\right.$$ (2)

in which Γ is a constant denoting the effect of IFN in reducing infectivity in target cells. To minimize the number of unknown parameters, we simplify the model by making the quasi-steady-state assumption that the dynamics of IFN are much faster than the dynamics of productively infected cells. This approach aligns with methodologies used in many studies, such as [26, 41]. Thus $\frac{dF}{dt}=0$, which gives $sI_{2}=\rho F$. Let $\gamma =\Gamma \frac{s}{\rho }$, then model (2) becomes


$$\left\{ \begin{array}{c} \frac{dT}{dt}=\lambda_{1}-d_{1}T-\frac{\beta }{1+\gamma I_{2}}TV,\\ \frac{dI_{1}}{dt}=\frac{\beta }{1+\gamma I_{2}}TV-kI_{1},\\ \frac{dI_{2}}{dt}=kI_{1}-\delta I_{2},\\ \frac{dV}{dt}=pI_{2}-c_{0}V. \end{array}\right.$$ (3)

We call this model the innate model throughout the text.

The second extended model considers adaptive immune response. All 18 animals measured significant T cell activation and antibody responses against mpox virus on day 7 and day 14 post-infection, respectively [35]. Recall that viral DNA peaked on day 7 or 10 following mpox infection in the experimental macaques [35]. This indicates the important role of adaptive immune response in decreasing viral load. Due to the lack of data on the adaptive immune response, we consider nonspecific adaptive immune response in this paper. That is, we assume that the adaptive immune response is activated on day τ and the death rate of virus increases from the base rate *c*_0_, following a revised sigmoid function $\frac{c_{*}}{1+e^{-(t-\mu \tau )}}$, where c_*_ represents the maximum impact of the immune response on reducing the viral load, and μ dictates how fast the immune response increases. Based on the above description, the mpox infection model with adaptive immune response is:


$$\left\{ \begin{array}{c} \frac{dT}{dt}=\lambda_{1}-d_{1}T-\beta TV,\\ \frac{dI_{1}}{dt}=\beta TV-kI_{1},\\ \frac{dI_{2}}{dt}=kI_{1}-\delta I_{2},\\ \frac{dV}{dt}=pI_{2}-c(t)V, \end{array}\right.$$ (4)

where


$$c(t)=\left\{ \begin{array}{cc} c_{0} & t\leq \tau ,\\ c_{0}+\frac{c_{*}}{1+e^{-(t-\mu \tau )}} & t>\tau , \end{array}\right.$$ (5)

represents the nonspecific adaptive immune response inhibiting MPXV. This model is termed the adaptive model throughout this study.

The third extended model includes both innate and adaptive immune responses. The ODEs for the model are:


$$\left\{ \begin{array}{c} \frac{dT}{dt}=\lambda_{1}-d_{1}T-\frac{\beta }{1+\gamma I_{2}}TV,\\ \frac{dI_{1}}{dt}=\frac{\beta }{1+\gamma I_{2}}TV-kI_{1},\\ \frac{dI_{2}}{dt}=kI_{1}-\delta I_{2},\\ \frac{dV}{dt}=pI_{2}-c(t)V. \end{array}\right.$$ (6)

We call this model the full model throughout the text.

### The model with antiviral tecovirimat treatment

We extend model (6) by including the effect of tecovirimat, as it is the most commonly used antiviral drug for treating mpox infection, which was primarily developed and used to treat smallpox [15]. Let ϵ be the effectiveness of tecovirimat, which is between 0 and 1. Note that ϵ=1 indicates that tecovirimat is 100% effective in preventing infected cells from producing new infectious MPXV. The modified model with tecovirimat is described by


$$\left\{ \begin{array}{c} \frac{dT}{dt}=\lambda_{1}-d_{1}T-\frac{\beta }{1+\gamma I_{2}}TV,\\ \frac{dI_{1}}{dt}=\frac{\beta }{1+\gamma I_{2}}TV-kI_{1},\\ \frac{dI_{2}}{dt}=kI_{1}-\delta I_{2},\\ \frac{dV}{dt}=(1-\epsilon )pI_{2}-c(t)V,\\ \frac{dV_{NI}}{dt}=\epsilon pI_{2}-c(t)V_{NI}, \end{array}\right.$$ (7)

where $V_{NI}$ is sequestered infectious viruses or non-infectious viruses generated due to the effect of tecovirimat. Note that model (7) only holds following treatment initiation (not necessarily immediately following infection). All other parameters and variables are the same as those in model (6).

### Parameter identification

The life span of human monocytes is approximately 4 to 7 days [42, 43], thus the death rate of uninfected monocytes *d*_1_ is assumed to be $0.15\;{\text{day}}^{-1}$. In [44], Kim *et al* calculated that the total number of monocytes for a healthy adult rhesus macaque is approximately 372 ± 170 counts/μl. This fact allows us to fix the density of monocytes in the absence of infection *T*_0_ to $2\times 10^{5}$ counts/ml. Thus the recruitment rate of uninfected monocytes is $\lambda_{1}=2\times 10^{5}\times 0.15=$ 3 × $10^{4}\;{\text{ml}}^{-1}{\text{day}}^{-1}$ from the steady-state of target cells before infection. Cell culture trials have demonstrated that high levels of mpox viral titer were detectable 4-8 hours post infection [45–47]. We set the eclipse period $k=4\;{\text{day}}^{-1}$ (corresponding to $\frac{1}{k}=6$ hours) according to [47], which indicates that this time point allows for starting the first replication but to avoid the risk of next cycle. For the clearance rate of free virus, we assume $c_{0}=10\;{\text{day}}^{-1}$ as in vivo viral clearance is usually fast in many infection, including for respiratory infections such as SARS-CoV-2 and influenza [20, 48]. The initial number of infected cells (either in an eclipse phase *I*_1_(0) or in the productive phase *I*_2_(0)) are set to 0 counts/ml. The initial viral load *V*(0) is chosen to be 50 copies/ml since it is the first recorded data point for all 18 macaques [35].

From the expression of *c*(*t*), it is clear that μ determines the temporal location and smoothness of the sigmoidal transition for *c*(*t*). As shown in the S1 Fig, when μ≤1, the function exhibits an immediate and sharp increase at the activation time τ. For μ>1, the growth becomes smoother, with larger values of μ leading to a delayed onset of significant growth. To simulate a moderate delay in the activation of the adaptive immune response while striking a balance between activation timing and smooth progression, we set μ=1.7. A sensitivity analysis, conducted in the following section by varying μ within the range [0.5,2.5], demonstrates that model results are robust to the variations in μ. As shown in the S1 Fig, prior to the activation of nonspecific adaptive immune response (t≤τ), *c*(*t*) =10, which is the base clearance rate of virus (*c*_0_). As time *t* is greater than τ, the value of *c*(*t*) begins to increase and eventually stabilizes at its maximum value of $c_{0}+c_{*}$. μ is dimensionless because it serves as a scaling factor, ensuring the argument of the exponential term of *c*(*t*) is unitless.

**Fig 2:**
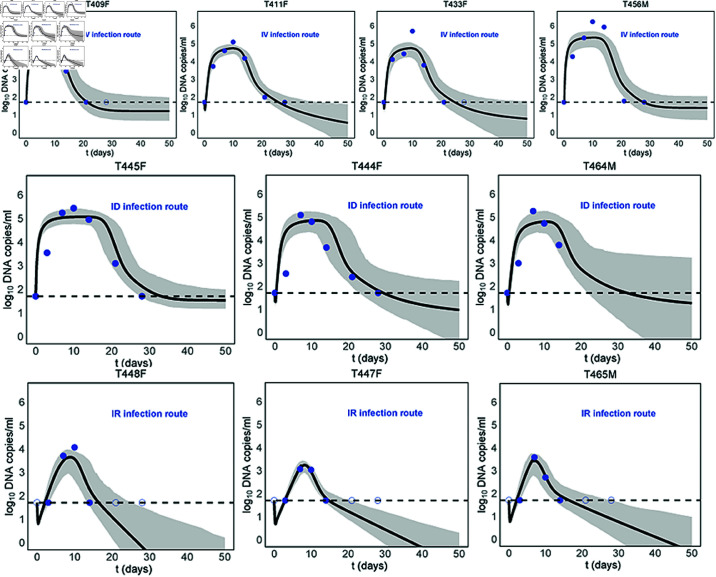
Full model (6) fitting to viral load data of 10 macaques infected with the same dose of MPXV. The blue symbols (dots and open circles) represent the data on the log_10_-scale, in which the open circles indicate viral loads below the detection limit (50 DNA copies/ml). The black solid lines are the model predictions, while the horizontal dashed lines represent the viral load detection limit. The best-fit parameter estimates are listed in Table 2. The shaded regions represent 95% confidence interval (CI) of the model simulation. Model (6) provides a better fit and shows that the viral load decay has two phase, with the slopes of these phases being impacted by the infection route.

The remaining parameters of models (1) to (6) are estimated by fitting models to the viral load data reported in [35]. Note that in [35], researchers collected viral load data for each macaque at days 0, 3, 7, 10, 14, 21, and 28 following infection. The real time polymerase chain reaction (PCR) method used in their study has a detection limit of 50 DNA copies/ml. This implies that the viral DNA concentrations below this threshold cannot be detected or reported accurately. Therefore, except for the first data point below the detection limit, subsequent data below this threshold are excluded from our data fit process, but are presented as blue open circles in Fig 2 and S2, S3 Figs. This is a commonly used practice in many studies [49]. Data fitting was performed using least square method and Bayesian inference, implemented in the Matlab 2020R and R programming language. Details of fitting process can be found in S1 text. Table 1 summarizes all model parameters and presents the ranges of fixed parameters used for sensitivity analysis (S4 and S5 Figs).

**Table 1:** Descriptions and values of parameters in model (6)

Parameter	Value	Description	Ranges
*V*(0)	50 (copies/ml)	Initial viral load	[10, 50]
$\lambda_{1}$	3$\times 10^{4}$ (${\text{ml}}^{-1}\cdot {\text{day}}^{-1}$)	Production rate of uninfected cells	[10^4^, 5$\times 10^{4}$]
*d*_1_	0.15 (${\text{day}}^{-1}$)	Death rate of uninfected cells	[0.05, 0.3]
*k*	4 (${\text{day}}^{-1}$)	1/Eclipse period	[2, 6]
*c*_0_	10 (${\text{day}}^{-1}$)	Viral clearance rate	[5, 20]
μ	1.7	Rate of immune activation	[0.5, 2.5]
γ	Fitted	Effect of IFN in reducing infectivity of target cells	
c_*_	Fitted (${\text{day}}^{-1}$)	Maximum effect of nonspecific adaptive immune response	
τ	Fitted (days)	Activated time of nonspecific adaptive immune response	
β	Fitted ($\text{ml}\cdot {\text{day}}^{-1}$)	Infection rate of cells by cell-free infection	
δ	Fitted (${\text{day}}^{-1}$)	Death rate of productively infected cells	
*p*	Fitted (${\text{day}}^{-1}$)	Production rate of viruses	

**Table 2:** Best fit parameter estimates of the model (6) for each macaque and their 95% CI

Dose & route	Macaque	β ($\text{ml}\cdot {\text{day}}^{-1}$)	δ (${\text{day}}^{-1}$)	*p* (${\text{day}}^{-1}$)	γ	τ (days)	$c_{*}$
**10^6^ TCID50 IV**	T409F	5.12 [3.22-7.02]$\times 10^{-6}$	0.41 [0.14-0.73]	1280 [774-1783]	0.96 [0.39-1.57]	10 [8-12]	467 [193-752]
	T411F	6.14 [2.70-10.28]$\times 10^{-7}$	0.32 [0.16-0.54]	1076 [633-1585]	0.07 [0.04-0.11]	9 [7-10]	466 [211-756]
	T433F	11.01 [3.85-1.96]$\times 10^{-7}$	0.33 [0.16-0.51]	858 [430-1335]	0.08 [0.02-0.14]	10 [8-12]	490 [226-769]
	T456M	11.05 [3.48-19.86]$\times 10^{-6}$	0.39 [0.21-0.56]	884 [446-1386]	0.19 [0.008-0.31]	11 [9-13]	874 [373-1021]
	**Mean**	4.46$\times 10^{-6}$	0.36	1025	0.33	10	574
	**SD**	4.81$\times 10^{-6}$	0.04	196	0.43	0.82	200
**10^5^ TCID50 IV**	T437F	4.14 [2.49-5.96]$\times 10^{-7}$	0.41 [0.24-0.58]	1088 [655-1567]	0.04 [0.009-0.07]	8 [7-9]	338 [173-519]
	T438F	3.25 [1.81-5.02]$\times 10^{-7}$	0.41 [0.21-0.62]	1281 [813-1788]	0.05 [0.01-0.09]	9 [8-12]	327 [157-512]
	T439F	2.06 [1.23-2.98]$\times 10^{-7}$	0.21 [0.12-0.3]	1003 [655-1427]	0.33 [0.16-0.51]	11 [9-13]	319 [149-505]
	T461M	10.43 [3.51-18.93]$\times 10^{-7}$	0.39 [0.13-0.71]	975 [522-1482]	0.13 [0.06-0.21]	10 [8-12]	327 [148-512]
	**Mean**	4.96$\times 10^{-7}$	0.36	1087	0.14	10	328
	**SD**	3.72$\times 10^{-7}$	0.09	138	0.13	1	8
**10^4^ TCID50 IV**	T441F	8.02 [6.20-9.94]$\times 10^{-8}$	0.19 [0.03-0.36]	519 [360-715]	0.06 [0.03-0.1]	10 [7-12]	88 [38-143]
	T442F	6.83 [4.91-8.76]$\times 10^{-7}$	0.99 [0.46-1.53]	387 [225-587]	0.99 [0.48-1.54]	20 [16-27]	256 [62-447]
	T443F	5.19 [3.65-6.88]$\times 10^{-8}$	0.32 [0.14-0.50]	1283 [901-1712]	0.01 [0.002-0.02]	11 [9-13]	372 [193-553]
	T462M	9.22 [7.32-11.09]$\times 10^{-8}$	0.26 [0.08-0.42]	1310 [906-1713]	0.06 [0.02-0.09]	10 [8-12]	334 [168-512]
	**Mean**	2.27$\times 10^{-7}$	0.44	875	0.28	13	263
	**SD**	3.05$\times 10^{-7}$	0.38	490	0.48	5	126
**10^6^ TCID50 ID**	T444F	7.79 [5.74 - 9.84]$\times 10^{-7}$	0.32 [0.09-0.62]	1021 [484-1653]	0.07 [0.02-0.1]	9 [6-12]	442 [173-733]
	T445F	4.88 [3.03-6.91]$\times 10^{-4}$	0.35 [0.14-0.59]	122 [84-159]	0.32 [0.15-0.52]	11 [9-13]	599 [502-699]
	T464M	5.75 [3.67-7.75]$\times 10^{-7}$	0.32 [0.03-0.7]	934 [567-1315]	0.04 [0.01-0.08]	9 [6-11]	290 [91-486]
	**Mean**	1.63$\times 10^{-4}$	0.33	692	0.14	9	443
	**SD**	2.81$\times 10^{-4}$	0.017	495	0.15	1.15	154
**10^6^ TCID50 IR**	T447F	6.10 [4.58-7.78]$\times 10^{-8}$	0.22 [0.13-0.31]	1203 [900-1594]	0.03 [0.01-0.05]	7 [6-8]	376 [182-582]
	T448F	9.03 [7.2-10.92]$\times 10^{-8}$	0.51 [0.24-0.79]	1263 [856-1713]	0.05 [0.02-0.08]	8 [6-11]	158 [67-257]
	T465M	8.82 [6.90-10.75]$\times 10^{-8}$	0.21 [0.11-0.31]	1085 [729-1542]	0.04 [0.02-0.06]	7 [5-8]	323 [145-512]
	**Mean**	7.98$\times 10^{-8}$	0.31	1184	0.04	7	286
	**SD**	1.63$\times 10^{-8}$	0.17	91	0.01	0.58	114

Note: TCID50 is a unit that represents 50% tissue culture infective dose

## Results

### Viral dynamics of mpox infection

We use four mathematical models to fit the viral load data of mpox infection in 18 macaques from [35], respectively. Detailed descriptions of these models are provided in the “ **Materials and methods**" section and the specifics of the data-fitting process are outlined in the S1 text.

To directly compare the viral dynamics across three infection routes, we present the results for 10 macaques infected with the same dose of 10^6^ TCID50 MPXV in a single figure. This includes 4 macaques infected via the IV route, 3 macaques via the ID route, and 3 macaques via the IR route. Fig 2 presents the data-fitting results for the full model (6), while S2 Fig summarizes the results for the basic model (1), innate model (3) and adaptive model (4). The best fits to the remaining 8 macaques, which are infected with 10^5^ TCID50 and 10^4^ TCID50 doses MPXV via IV route, are shown in the S3 Fig.

Overall, the basic model (1) and the innate model (3) predict a sustained viral load across all 10 macaques, with the innate model stabilizing at a lower level than the basic model (S2 Fig). Although there is a natural clearance of virus in the model (1), the constant recruitment rate of susceptible cells contributes to viral load persistence. The innate immune response can suppress the viral replication to a lower level; however, it fails to achieve complete viral clearance, reflecting its limitations in resolving the infection independently. In contrast, the adaptive model (4) effectively captures the observed viral dynamics, including the initial peak and the subsequent decline in viral load (S2 Fig). The full model (6), which integrates both innate and adaptive immune responses, provides a closer alignment with the observed data and also describes the viral load dynamics (Fig 2). To determine which one of these two models provides a better fit, we calculate the Akaike Information Criterion (AIC), a standard metric for evaluating model fit and parsimony. A lower AIC value indicates a more parsimonious model with stronger explanatory power. The results suggest that the full model gives a better fit to the viral load data from 18 infected macaques (see S1 Table). This indicates that both innate and adaptive immune response play critical roles in shaping the MPXV dynamics.

From Fig 2, we observe that the model simulations of different infection routes vary significantly. In the cases of IV and ID infection routes (the first two rows in Fig 2), the viral loads initially increase to the peak level. Following the peak, the viral load in all macaques declines below the detection limit (50 DNA copies/ml) in two phases. The slope of decline in the first phase is steeper than that in the second phase. This might be due to the use of a revised sigmoid function to describe the effects of non-specific adaptive immune responses (“ **Materials and methods**"). Once the adaptive immune responses are activated, they trigger a dramatic decrease in viral load in the first phase. As the immune response levels reach their maximum, a lower decline in viral load occurs in the second phase. We should note that for the macaque T464M, the data for days 21 and 28 post-infection are missing for unspecified reasons. The viral dynamics of mpox infection in the case of the IR infection route differ significantly from those of the previous two infection routes. The viral load initially declines slightly and then increases to the peak value (the third row in Fig 2). Further, the decay rate of the second phase remains rapid, suggesting persistently active immune responses.

The best fit parameter estimates of the model (6) are shown in Table 2. Surprisingly, the infection rate β is notably small through the IR route, consequently resulting in low levels of both innate and adaptive immune responses (γ and c_*_). However, the activated time of nonspecific adaptive immune responses via this infection route is earliest (τ). This is reasonable because the rectal mucosal environment might exhibit higher levels of immune activity due to potential injuries during injection, which mimics the sexual contact among MSM [50]. Compared to sexual contact, viral infection through skin-to-skin contact (ID infection route) may be more effective due to the significant higher infection rate, which elicits higher levels of immune responses.

### Infectivity of each macaque

The transmission mode of mpox in the 2022 outbreak significantly differed from the traditional mode, primarily affecting the MSM community [2]. We try to uncover reasons and features behind this phenomenon by calculating the infectiousness of an individual post-infection. Here and in what follows, infectiousness is defined as the probability that an infected individual transmits one or more infectious virions during exposure, resulting in successful infection in a susceptible individual [26, 51]. In the following, we calculate the infectiousness of each infected macaque post infection by using our best-fit model (6), following the work of [26, 52, 53]. Transmission routes considered here are direct blood contact, skin-to-skin and sexual contact, in accordance with the experiment conducted by Aid *et al*. [35].

During each contact, an infected individual transfers both infectious and non-infectious virions and only the infectious virions induce the new infection (Fig 1A). We first need to determine the ratio of infectious virions per contact, that is, the relationship between infectious virions ($V_{infectiou}$) and total viral load (*VL*) in a sample. Assuming that the number of $V_{infectious}$, *X*, in an experimental sample, is a random variable obeying the Poisson distribution with parameter Λ=E(X), in which *E*(*X*) is the average number of infectious viruses. In the following, we use three different models to describe the relationship between the average number of infectious viruses and total viral load.

(1) The linear model


$$E(X)=A_{1}\cdot VL,$$

where *A*_1_ is the constant. This means that the average number of infectious viruses is simply proportional to the total viral load in a sample.

(2) The power-law model


$$E(X)=A_{2}\cdot VL^{B_{2}},$$

where *A*_2_ and *B*_2_ are constant parameters.

(3) The saturation model


$$E(X)=A_{3}\cdot \frac{VL}{VL+B_{3}},$$

where *A*_3_ and *B*_3_ are constant parameters. We will fit each of the three models to the data to determine the most suitable one.

In the study [54], the quantitation cycle (Cq) value and the infectious virus titre were measured from each sample in cell culture by using real-time PCR and plaque assay in a patient sample, respectively. The Cq value, also known as the threshold cycle value, is the cycle number at which the fluorescence generated within a reaction crosses the threshold and becomes detectable above the background signal in PCR. Then the *VL* can be determined by using the following linear equation


$$Cq=-mlog_{10}(VL)+n,$$ (8)

where *m* and *n* are positive constants.

**Fig 3:**
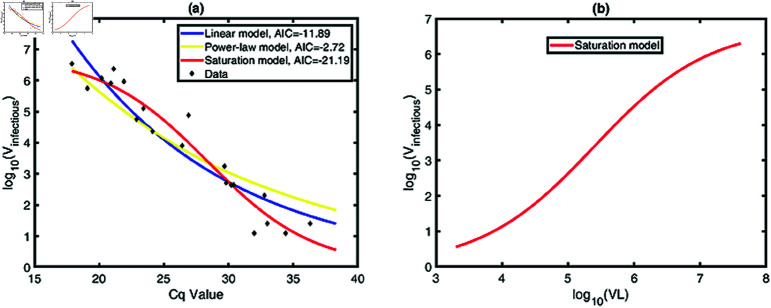
The relationship between host infectivity and *VL.* (a) Best fits of three models to the dataset (black squares) from [54]. The blue line, yellow line and red line represent the linear model, power-law model and saturation model, respectively. According to the AICc values, the saturation model describe the data well, with the best-fit parameters *A*3 = 6.78, $B3=5\times 10^{4}$, *m* = 9.5, *n* = 72.87. (b) The predicted relationship between infectious virions ($V_{infectious}$) and total viral load (*VL*) in log_10_-scale.

We fit each of three models to the dataset collected in [54]. To compare the results with different models, we calculate the values of corrected Akaike Information Criterion (AICc), given by


$$AICc=u\ln\left(\sum_{i=1}^{n}(\tilde{log_{10}(V_{infectious}^{i}})-log_{10}(E(V_{infectious}^{i})){)}^{2}/u\right)+2w+\frac{2w(w+1)}{u-w-1},\;i=1,2,\cdots ,K.$$ (9)

where $\tilde{V_{infectious}^{i}}$ is the data of infectious virus titre at the *i*th point from [54], $E(V_{infectious}^{i})$ is the corresponding values predicted by the model, *u* is the number of data points and *w* is the number of estimated parameters. Note that a lower AICc value suggests a better model. The results are shown in the Fig 3A. According to the AICc values, the saturation model is the best model to describe the dataset in [54]. Thus, we use the saturation model to depict the relationship between the infectious virus and total viral load in a sample, which is shown in the Fig 3B.

**Fig 4:**
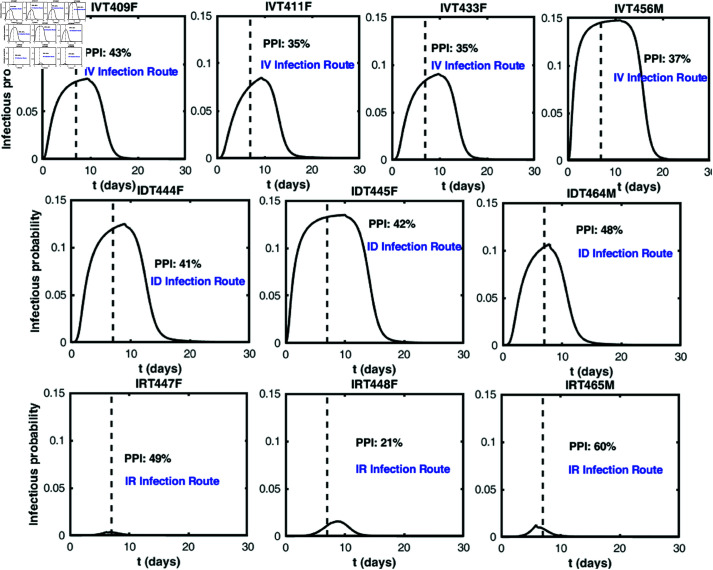
The infectivity of infected macaques. The infectious probability of 10 macaques post-infection with MPXV, which is calculated by using both model (6) with parameters listed in Tables 1 and 2, as well as the saturation model with best-fit parameters. PPI stands for proportion of presymptomatic infectiousness and the vertical dashed lines denote symptom onset.

Now we can calculate the infectiousness of an individual following infection. During per contact at time *t*, each infectious virion either transmits successfully or fails to do so. This process can be regarded as a Bernoulli trial. We assume the probability of successful transmission for each virion is θ(t) and then the probability of transmitting *l* infectious virions, {*f*_*l*_}, follows a Binomial distribution, fl=(nl)θ(t)l(1−θ(t))n−l, where *n* is the total number of virions [55]. When *n* is very large and θ(t) is very small, the probability of successful transmission is Poisson distributed and (1 − θ(t)) is the probability of waiting time until the first successful transmission. Then the occurrence with at least one successful transmission follows an exponential distribution, which means that


$$\theta (t)=1-exp(-\phi \cdot \frac{VL(t)}{VL(t)+B_{3}}),$$ (10)

where ϕ is a constant depending on the contact mode. Note that the maximum probability is 1 − exp(−ϕ), which approximates to ϕ if ϕ is small. According to an epidemiological review [56], the secondary attack rate for mpox infections varies by context, but is generally lower than 20%. Thus we set ϕ to 0.2 so that the maximum transmission probability per contact is about 20%. Using the best-fit parameter value $B_{3}=5\times 10^{4}$ DNA copies/ml, we calculate the infectiousness of each macaque. As shown in Fig 4, the infectiousness initially increases to a maximum and then decreases over time. As stated in [35], clinical signs were observed on day 7 post-infection in all infected 18 macaques. To determine the percent infectiousness that is presymptomatic, we divide the area under the infectious probability curve before day 7 by the entire area under the infectious probability curve. We find that the proportion of presymptomatic infectiousness is relatively high in both infections occurring through skin-to-skin contact (ID infection route) and sexual contact (IR infection route). This suggests that presymptomatic transmission contributes significantly to the spread of mpox transmission in the current epidemic.

### Impact of infection routes on host immunity and infectivity

We study how the viral dynamics and infectiousness vary with time across three infection routes under varying assumptions. To focus on the impact of infection routes, we use data from 10 macaques infected with the same dose of 10^6^ TCID50 MPXV. The observations among the macaques infected via same routes show consistent patterns. Thus in the main text, we present the macaques T409F (IV infection route, IVT409F), T464M (ID infection route, IDT464M) and T465M (IR infection route, IRT465M) as representative examples for the three infection routes. The similar plots for the remaining macaques are provided in the S6–S10 Figs.

**Fig 5:**
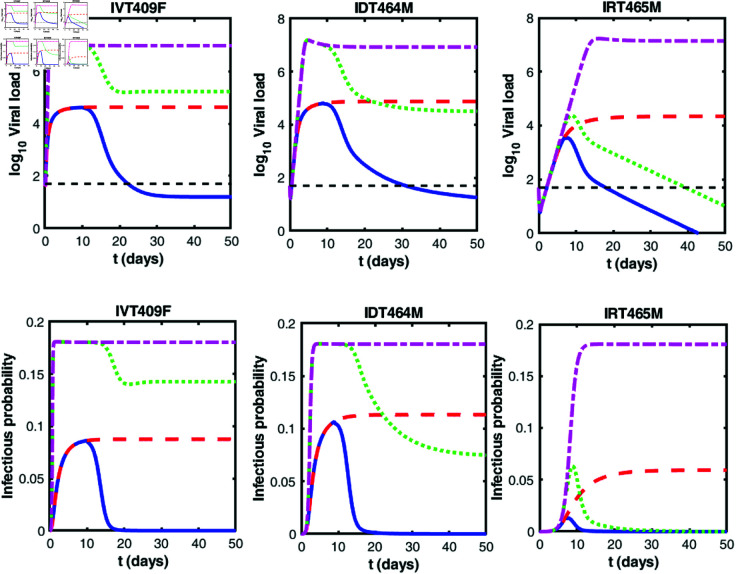
Dynamics of virus and infectious probability. Virus dynamics (upper row) and infectious probability (bottom row) over time under different scenarios for three infection routes, i.e., IV infection route (IVT409F), ID infection route (T464M) and IR infection route (IRT465M). The blue solid line represents the scenario with both innate and adaptive immune responses; the green dotted line corresponds to the case with adaptive immune response alone; the red dashed line depicts the case with innate immune response alone; and the magenta dashed-dotted line illustrates the case without any immune response. Black dashed lines represent the viral load detection limit (50 DNA copies/ml). The innate or adaptive immune response, if excluded, its corresponding parameter, γ or c_*_, is assumed to be 0, while the remaining parameters are consistent with those used in Fig 4.

Fig 5 presents the dynamics of MPXV and infectious probability under different immunity scenarios. The upper row is for *log*_10_ viral load while the bottom row is for infectious probability. Across all three infection routes, the presence of both innate and adaptive immunity, the viral loads peak around day 7–10. This agrees with the clinical data collected in [35]. In the absence of immunity, the viral loads in three infection routes increase rapidly and remain at high levels (the magenta dashed-dotted lines in upper panels). When only one type of immunity exists, the dynamics of viral load under the three infection routes are very different. For the IV and ID infection cases, whether innate or adaptive immunity is present alone, the viral load can not be eliminated. In contrast, when considering the IR infection route, adaptive immunity alone is effective in reducing the viral load below the detection limit (the green dotted line in IRT465M). Note that the virus dynamics under the assumptions of no immunity, innate immunity only and adaptive immunity only differ from those presented in the S2 Fig, as they are based on different parameter estimation approaches. In this figure, we are using parameter estimates obtained from the full model, with specific immune parameters (γ and c_*_) set to zero for each scenario. In contrast, S2 Fig represents the direct data fitting results for each individual model (basic model, innate model, and adaptive model), where parameters are independently estimated for the respective models. Consequently, while both figures aim to illustrate the effects of different immune responses, the differing parameter estimation methodologies lead to different model predictions.

Taken together, both the mechanism and the effectiveness of the host immunity vary significantly depending on the infection route. These observations strongly agree with those found in experiment (Fig 3 in [35]). Similar phenomena can be observed in the dynamics of infectiousness of three infection routes. In addition, we find that infectiousness is maximized near the peak viral load. Thus in the following we test the impact of various potential therapies aimed at reducing the viral load peak in infected individuals, thereby decreasing their infectiousness.

### Early antiviral treatment may reduce infectiousness across infection routes

**Fig 6:**
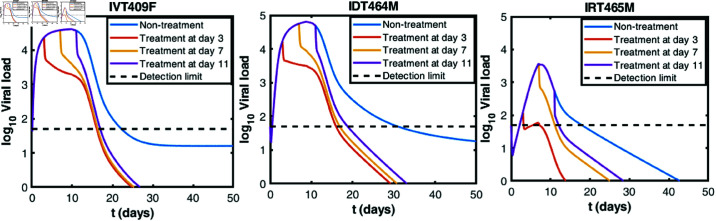
Virus dynamics under drug treatment. Predicted virus dynamics with and without treatment for three infection routes, assuming treatment starts at day 3, 7, and 11 post-infection, respectively. Black dashed lines represent the viral load detection limit (50 DNA copies/ml). The drug efficacy ϵ is fixed to be 0.8.

**Fig 7:**
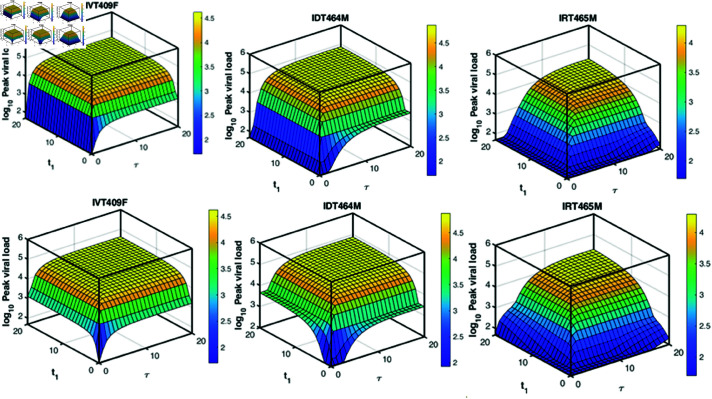
The peak viral load changes with the timing of drug treatment (*t*_1_) and the activation of adaptive immune response (τ) for three infection routes, respectively. The first row shows results under normal adaptive immunity, while the second row represents compromised adaptive immunity (c_*_ reduced by 90%). All other parameters are consistent with Fig 6.

With using the extension model (7) including tecovirimat treatment (“ **Materials and methods**"), we study the viral dynamics under drug therapy, as shown in Fig 6. The viral load discussed here and below refers to *V*, as defined in the forth equation of model (7). The efficacy of tecovirimat in controlling human mpox virus replication remains under investigation. However, tecovirimat has been shown to provide full protection in animal models against mpox infection and is generally well-tolerated in humans [15, 16, 57]. Due to the lack of pharmacokinetic data, we assume a constant efficacy of 80%, which suggests substantial effectiveness but are not comprehensive. It is administrated starting at days 3, 7, 11 post-infection, respectively. We find that early treatment can shorten the time to viral clearance and the lower the peak level of viral load across all three infection routes. However, initiating tecovirimat treatment before the activation of adaptive immune responses results in a plateau phase, while after immune activation, the viral loads rapidly decrease. We hypothesize that there might be an interplay between the adaptive immune responses and tecovirimat. Therefore, we investigate the effect of time of tecovirimat initiation (*t*_1_) and time of immune activation (τ) on the peak viral load, as shown in Fig 7.

The strength of adaptive immune response (c_*_), if activated, is set to its normal value in the first row of Fig 7, based on parameter estimates derived from data fitting (Table 2). In the second row, c_*_ is reduced by 90%, representing a compromised immunity. For IV infection (IVT409F), under normal c_*_, the peak viral load consistently increases as the time required for immune activation extends. However, as *t*_1_ decreases, the rise in peak viral load becomes less pronounced, indicating a reduced effect of treatment timing on viral suppression with earlier intervention. This suggests that early treatment not only lowers the peak viral load but also decreases the sensitivity of the system to the immune activation timing (τ). A similar pattern is observed in the dynamics of peak viral load in ID464M and IR465M. Notably, in the context of IR infection route (IR465M), the prompt initiation of drug treatment can effectively inhibit viral replication even with a delayed immune response.

**Fig 8:**
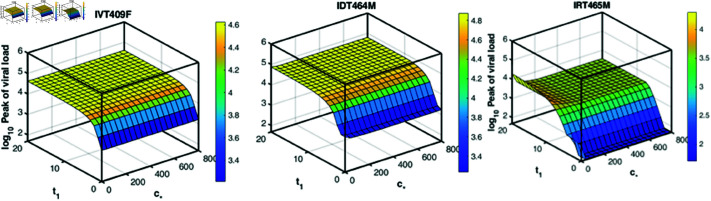
The peak viral load changes with the timing of drug treatment (*t*_1_) and the strength of adaptive immune response (c_*_) for three infection routes, respectively. The remaining parameters are the same as in Fig 6.

In the case of reduced c_*_ (second row), across all three infection routes, the peak viral load is slightly elevated, with the surface increasing more steeply, particularly for larger *t*_1_ and τ. Notably, in this case, early drug treatment is required to control viral replication even if the immune response is activated early. Fig 8 illustrates the combined effects of timing of drug treatment (*t*_1_) and adaptive immune response strength (c_*_) on the peak viral load across three infection routes. In all cases, earlier treatment and stronger immune responses reduce the viral load, although the magnitude of the effect varies. Specifically, the IR infection route demonstrates the greatest sensitivity to the timing of drug treatment, while the IV infection route is the least sensitive. Collectively, these findings underscore the importance of early tecovirimat administration in combating mpox infection, particularly in the context of the current epidemic.

## Discussion and conclusion

Although MPXV has been identified for several years, the macaque model remains an ideal system for understanding the pathogenesis of MPXV. Investigating in vivo viral dynamics is crucial for uncovering patterns of virus transmission and aiding in the development of effective control strategies. Despite MPXV being known for some time, its within-host viral dynamics have not been extensively studied. MPXV can invade the host through various routes, with skin-to-skin contact and sexual contact emerging as significant transmission pathways during the 2022 outbreak. To better understand these dynamics, we developed mathematical models to study MPXV behavior under different infection routes. Research has shown that the innate and adaptive immune responses to MPXV in infected macaques are, in most cases, very similar to those observed in humans [34]. Therefore, we calibrated our models using unique experimental data on viral load from infected macaques collected in [35], and then explored host immune mechanisms and features of mpox infection in the context of the 2022 outbreak.

We estimated the key parameters of the model, as summarized in Table 1. Notably, the viral infection rate via skin-to-skin contact was substantially higher than that of the other two infection routes. This suggests that skin-to-skin contact may be a more favorable route for viral transmission in the current outbreak. Secondly, we demonstrated that the host’s adaptive immune response plays a critical role in controlling mpox infection via sexual contact (Fig 5 and S2 Fig). We established the relationship between infectious virions and total viral load in an experimental sample. Interestingly, the infectious virions increase with increasing viral load as a saturation function (Fig 3). This observation aligns with findings from [26, 52], though additional datasets are needed to further validate its robustness. We employed this saturation relationship to explore the role of presymptomatic transmission under various infection routes, leading to several intriguing results that may shed light on certain characteristics of the 2022 outbreak.

The results indicate that individuals infected via sexual contact generally exhibit a lower infectious probability (Fig 4 (IRT447F, IRT448F, IRT465M)). In contrast, individuals infected through skin-to-skin contact exhibited relatively high infectious probability, comparable to those infected via direct blood contact (Fig 4). Note that the parameter ϕ was fixed at 0.2 when calculating the infectious probability; this composite parameter encompasses the proportion of infectious virions transmitted per contact and the likelihood of each virion successfully establishing a new infection. Consequently, ϕ is influenced by the mode of contact. For sexual contact, we increased ϕ due to the elevated risk of infectious virions being transmitted through residual bodily fluids. However, even with ϕ values of 0.4, 0.6, and 0.9, the infectious probability for sexual contact remained significantly lower than for the other two infection routes (see S11 Fig). Further, we observed a high proportion of presymptomatic transmissions resulting from individuals themselves infected by sexual and skin-to-skin contact. Based on these observations, we speculate that skin-to-skin contact during sexual activity was a primary route of viral transmission, with presymptomatic transmission playing a critical role in the spread of MPXV during the 2022 outbreak. Although it was previously believed that person-to-person transmission of orthopoxviruses primarily occurred through close contact with symptomatic cases [58, 59], our findings suggest otherwise. Note that the small calibration sample size might impact the results, particularly given substantial individual heterogeneity in viral dynamics [60]. More data, if available in the future, can be used to validate the robustness of the results. If only individuals with significant clinical symptoms were infectious, the 2022-2023 outbreak among MSM would likely not have occurred, because if they are visibly ill or have visible lesions, they will most likely not engage in sexual activity. Our results are consistent with a study conducted in the United Kingdom, which found that 53% of mpox transmission occurred during the presymptomatic period [61].

Our model simulations indicate that the infection route can influence the interplay between host immunity and antiviral drug therapy (Figs 6, 7 and 8). In the context of the current outbreak, early administration of tecovirimat is crucial for significantly reducing peak viral load and accelerating viral clearance. However, caution is necessary when applying this conclusion in a clinical setting, as it is based solely on our mathematical model and relies on specific assumptions, despite being calibrated with real experimental data. One of the key assumptions is that viral clearance is mediated by a piecewise function representing the non-specific adaptive immune response due to the lack of longitudinal immunological data. As such, the model captures the overall effect of adaptive immunity on viral clearance without distinguishing between cellular and humoral components. More comprehensive data on immune response dynamics would allow for the incorporation of more detailed immune mechanisms into the model. Another important assumption is that the efficacy of tecovirimat remains constant over time. This assumption may lead to an overestimation of the impact of early tecovirimat administration, as drug concentrations are expected to vary over time [62]. It is also assumed that the adaptive immune response under drug treatment is the same as in the absence of treatment. In reality, the suppressed viral dynamics under treatment could plausibly affect the development of immune response. However, to date, data on the pharmacodynamics of tecovirimat in mpox-infected patients are limited. We plan to further investigate these issues as pharmacodynamic data become available. Further, the models in this study do not explicitly distinguish between the two infectious forms of the virus, IMVs and EEVs. While this simplification provides a general understanding of viral dynamics, it may overlook the distinct roles these forms play in infection and transmission. Future models could incorporate these dynamics explicitly to provide a more detailed representation, particularly as more data becomes available.

In conclusion, this study developed a robust modeling framework to explore within-host MPXV dynamics, integrating experimental data for greater accuracy. We examined the interactions between host cells and MPXV across different infection routes, shedding light on the characteristics of current mpox transmission and providing recommendations for drug treatment. As mpox cases continue to rise, particularly among MSM, we strongly encourage individuals in this community to take proactive steps to reduce the risk of transmission by limiting close physical contact after potential exposure, even if no symptoms are present. Early identification of potential infection is crucial in preventing spread before symptoms appear. Moreover, individuals with compromised immunity, such as those who are HIV-positive, should consider initiating antiviral medication as soon as possible after exposure.

## Supporting information

S1 FigVisualization of c(t).(**a**) c(t) changes with different values of μ. We fix *c*_0_=10, τ=10, and c_*_=300. The dashed line, representing c(t)/2, highlights shifts in the onset and progression of the growth curve as μ changes. (**b**)μ is fixed at 1.7 to show how c(t) varies with time t(TIF)

S2 Fig Data fitting results for 10 infected macaques using models (1), (3) and (4). Each panel represents an individual macaque. The first row corresponds to macaques infected via the IV route, the second row to those infected via the ID route and the third row represents to those infected via IR route. Model predictions are shown as solid lines: orange for the basic model (1), yellow for the innate model (3), and blue for the adaptive model (4). The black dashed line is the viral load detection limit (50 DNA copies/ml). The blue dots represent the viral load data on the log10-scale used for data-fitting.
(TIF)

S3 Fig Data fitting results of the remaining 8 macaques for four models. These macaques were infected with viral doses different from those administered to the other 10 macaques. Each panel represents an individual macaque. The first two rows represent the data-fitting results for 8 macaques using models (1), (3) and (4). Model predictions are shown as solid lines: orange for the basic model (1), yellow for the innate model (3), and blue for the adaptive model (4). The last two rows display the data-fitting results for the same 8 macaques using the full model (6). In all panels, blue dots and open circles represent the viral load data on the log10-scale used for fitting. The black dashed line is the viral load detection limit (50 DNA copies/ml).
(TIF)

S4 FigSensitivity analysis of parameter estimates with respect to variations in fixed parameters.We conduct a sensitivity analysis to test the robustness of our parameter estimates with respect to the fixed parameters ($V(0),\;\lambda_{1},\;d_{1},\;k,\;c_{0},\;\mu$). Each subplot shows the estimated values of the parameters (β,δ,p,γ,τ, and c_*_) under different fixed parameter settings. Bars represent the range of estimates across five evenly spaced values for each fixed parameter. For each fixed parameter, five evenly spaced values are sampled within its ranges shown in Table 1 and the model is refitted to the combined viral load dataset from all 18 infected macaques. Across the scenarios examined, the estimates of key parameters, including β, δ, *p*, γ, τ, and c_*_, showed consistent trends. For instance, the values of β varied slightly but remained within the range of $0.2\times 10^{-5}$ to 10^−5^, while δ ranged from approximately 0.3 to 0.5. Similarly, other parameters, such as *p*, τ, and c_*_, exhibited minimal variations across different fixed parameter values. Notably, these fluctuations are centered around the mean estimated values for the 18 macaques (Table 1), further supporting the robustness and reliability of the parameter estimates.(TIF)

S5 FigSensitivity analysis on fixed parameters in viral dynamics.Each panel corresponds to a different fixed parameter being varied: V(0), $\lambda_{1}$, *d*_1_, *k*, *c*_0_, and μ. The individual lines within each panel represent viral dynamics for five evenly spaced values of the corresponding fixed parameter, as indicated in the legends. This figure shows the predicted viral dynamics over time with different values of fixed parameters. It is clear that fixed parameters have minimal influence on overall viral dynamics. These results indicate that the estimated parameters and predicted viral dynamic are robust against variations in the fixed parameter values.(TIF)

S6 FigSimilar plots to those in Fig 5 for the remaining 7 macaques.The blue solid line represents the scenario in which both innate and adaptive immune responses are included. The green dotted line corresponds to the case with only the adaptive immune response, the red dashed line depicts the case with only the innate immune response, and the magenta dash-dotted line illustrates the scenario without any immune response. The black dashed line indicates the detection limit of the viral load (50 DNA copies/ml). When a specific immune response is excluded, the corresponding parameter, γ for innate immunity or c_*_ for adaptive immunity, is set to zero.(TIF)

S7 FigSimilar plots to those in Fig 6 for the remaining 7 macaques.Predicted virus dynamics with and without treatment for three infection routes, assuming treatment starts at day 3, 7, and 11 post-infection, respectively. Black dashed lines represent the viral load detection limit (50 DNA copies/ml). The drug efficacy ϵ is fixed to be 0.8.(TIF)

S8 Fig Similar plots to those in the first row of Fig 7 for the remaining 7 macaques. (TIF)

S9 Fig Similar plots to those in the second row of Fig 7 for the remaining 7 macaques. (TIF)

S10 Fig Similar plots to those in Fig 8 for the remaining 7 macaques. (TIF)

S11 FigSensitivity of infectiousness with respect to the maximum transmission probability per contact ϕ.Different colors correspond to distinct ϕ values: orange (ϕ=0.2), blue (ϕ=0.4), green (ϕ=0.6), and red (ϕ=0.9). The vertical dashed line marks the time of symptom onset.(TIF)

S1 Table Comparison of the best fit of the adaptive model and the full model using AIC values. (XLSX)

S1 Text Model data fitting and parameter estimation. (DOCX)
