# Supplementary figures and images for: Uncovering the impact of infection routes on within-host MPXV dynamics: Insights from a mathematical modeling study
Source: PLoS Comput Biol. 2025 May 19;21(5):e1013073. doi: 10.1371/journal.pcbi.1013073 (PMC12088049; doi:10.1371/journal.pcbi.1013073)

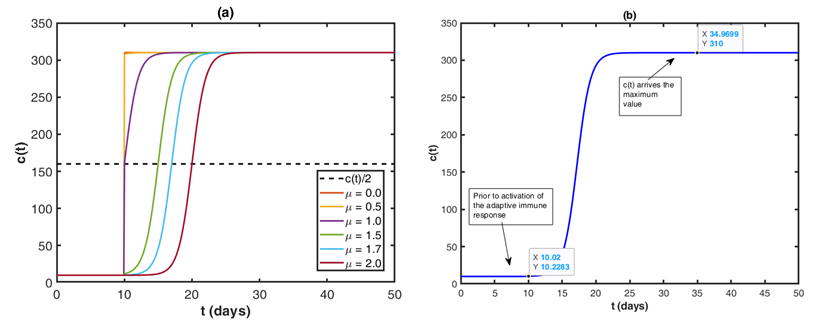

Supplement: S1 Fig — (a) c(t) changes with different values of μ. We fix c0=10, τ=10, and c*=300. The dashed line, representing c(t)/2, highlights shifts in the onset and progression of the growth curve as μ changes. (b)μ is fixed at 1.7 to show how c(t) varies with time t (TIF) [file pcbi.1013073.s001.tif]

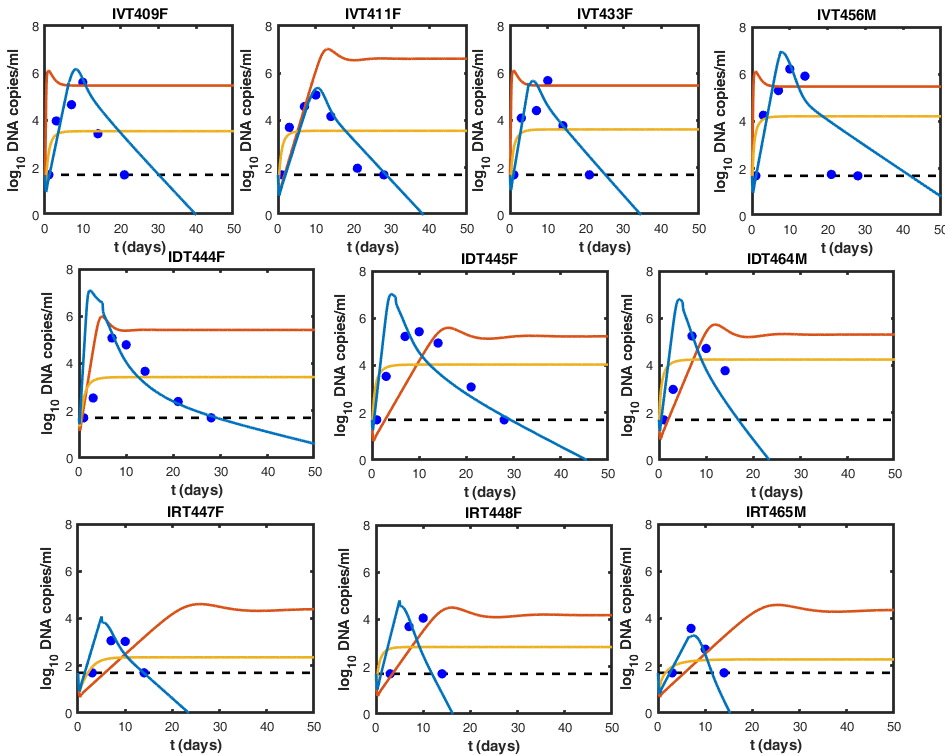

Supplement: S2 Fig — Each panel represents an individual macaque. The first row corresponds to macaques infected via the IV route, the second row to those infected via the ID route and the third row represents to those infected via IR route. Model predictions are shown as solid lines: orange for the basic model (1), yellow for the innate model (3), and blue for the adaptive model (4). The black dashed line is the viral load detection limit (50 DNA copies/ml). The blue dots represent the viral load data on the log10-scale used for data-fitting. (TIF) [file pcbi.1013073.s002.tif]

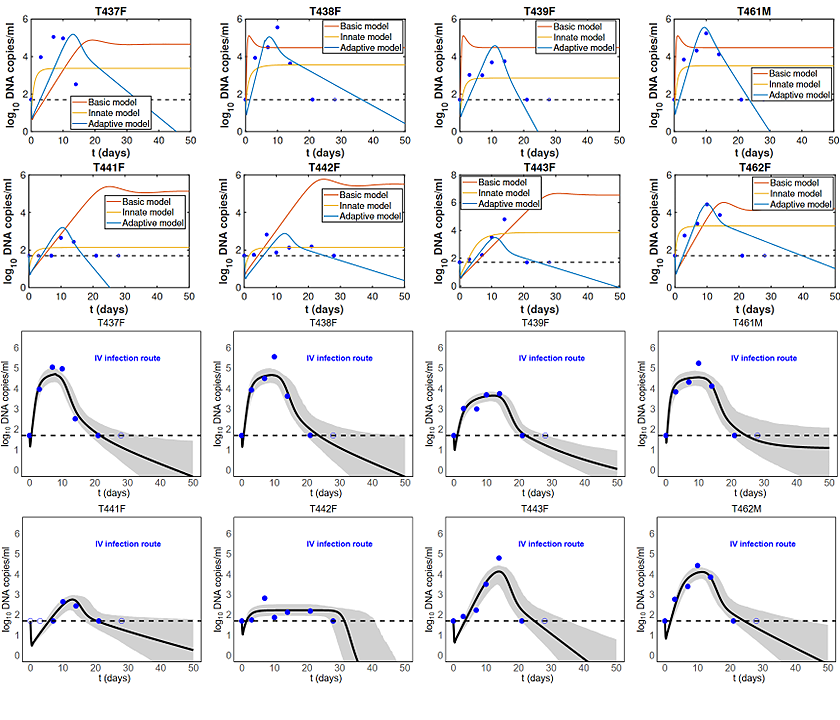

Supplement: S3 Fig — These macaques were infected with viral doses different from those administered to the other 10 macaques. Each panel represents an individual macaque. The first two rows represent the data-fitting results for 8 macaques using models (1), (3) and (4). Model predictions are shown as solid lines: orange for the basic model (1), yellow for the innate model (3), and blue for the adaptive model (4). The last two rows display the data-fitting results for the same 8 macaques using the full model (6). In all panels, blue dots and open circles represent the viral load data on the log10-scale used for fitting. The black dashed line is the viral load detection limit (50 DNA copies/ml). (TIF) [file pcbi.1013073.s003.tif]

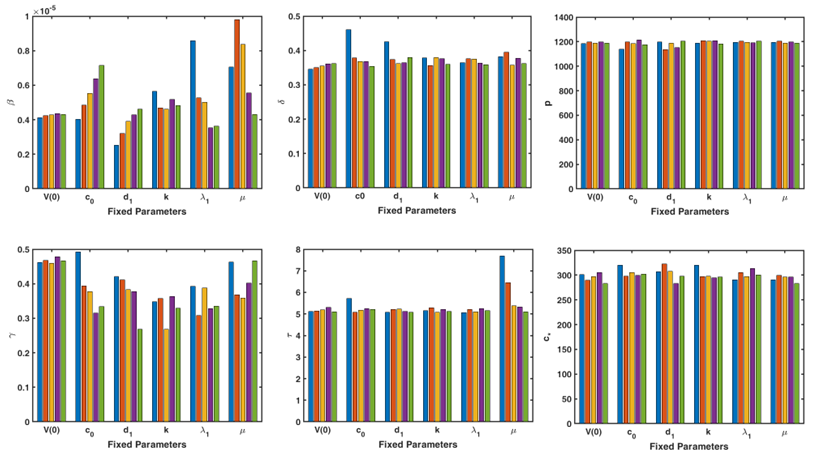

Supplement: S4 Fig — We conduct a sensitivity analysis to test the robustness of our parameter estimates with respect to the fixed parameters (V(0), λ1, d1, k, c0, μ). Each subplot shows the estimated values of the parameters (β,δ,p,γ,τ, and c*) under different fixed parameter settings. Bars represent the range of estimates across five evenly spaced values for each fixed parameter. For each fixed parameter, five evenly spaced values are sampled within its ranges shown in Table 1 and the model is refitted to the combined viral load dataset from all 18 infected macaques. Across the scenarios examined, the estimates of key parameters, including β, δ, p, γ, τ, and c*, showed consistent trends. For instance, the values of β varied slightly but remained within the range of 0.2×10^−5^ to 10−5, while δ ranged from approximately 0.3 to 0.5. Similarly, other parameters, such as p, τ, and c*, exhibited minimal variations across different fixed parameter values. Notably, these fluctuations are centered around the mean estimated values for the 18 macaques (Table 1), further supporting the robustness and reliability of the parameter estimates. (TIF) [file pcbi.1013073.s004.tif]

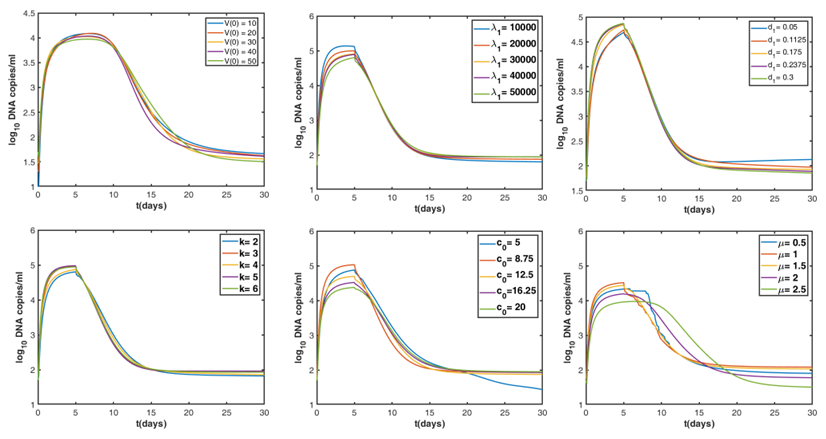

Supplement: S5 Fig — Each panel corresponds to a different fixed parameter being varied: V(0), λ1, d1, k, c0, and μ. The individual lines within each panel represent viral dynamics for five evenly spaced values of the corresponding fixed parameter, as indicated in the legends. This figure shows the predicted viral dynamics over time with different values of fixed parameters. It is clear that fixed parameters have minimal influence on overall viral dynamics. These results indicate that the estimated parameters and predicted viral dynamic are robust against variations in the fixed parameter values. (TIF) [file pcbi.1013073.s005.tif]

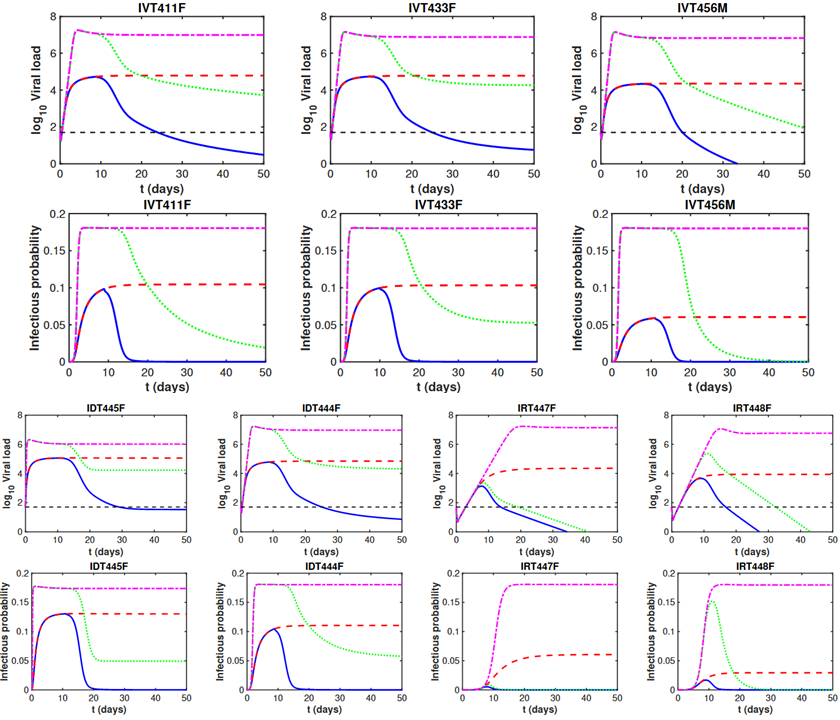

Supplement: S6 Fig — The blue solid line represents the scenario in which both innate and adaptive immune responses are included. The green dotted line corresponds to the case with only the adaptive immune response, the red dashed line depicts the case with only the innate immune response, and the magenta dash-dotted line illustrates the scenario without any immune response. The black dashed line indicates the detection limit of the viral load (50 DNA copies/ml). When a specific immune response is excluded, the corresponding parameter, γ for innate immunity or c* for adaptive immunity, is set to zero. (TIF) [file pcbi.1013073.s006.tif]

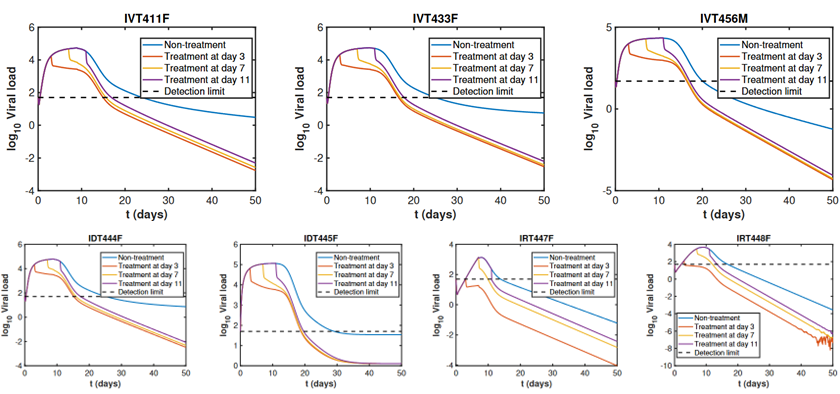

Supplement: S7 Fig — Predicted virus dynamics with and without treatment for three infection routes, assuming treatment starts at day 3, 7, and 11 post-infection, respectively. Black dashed lines represent the viral load detection limit (50 DNA copies/ml). The drug efficacy ϵ is fixed to be 0.8. (TIF) [file pcbi.1013073.s007.tif]

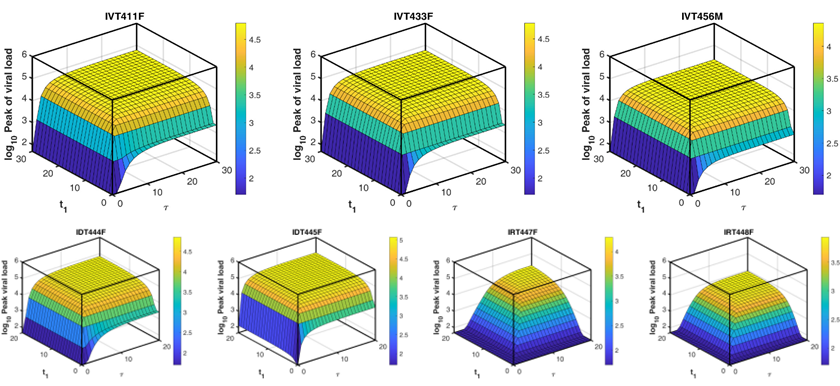

Supplement: S8 Fig — (TIF) [file pcbi.1013073.s008.tif]

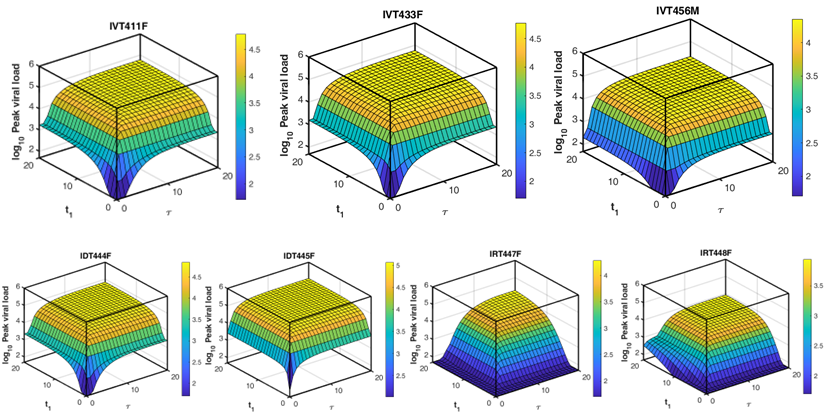

Supplement: S9 Fig — (TIF) [file pcbi.1013073.s009.tif]

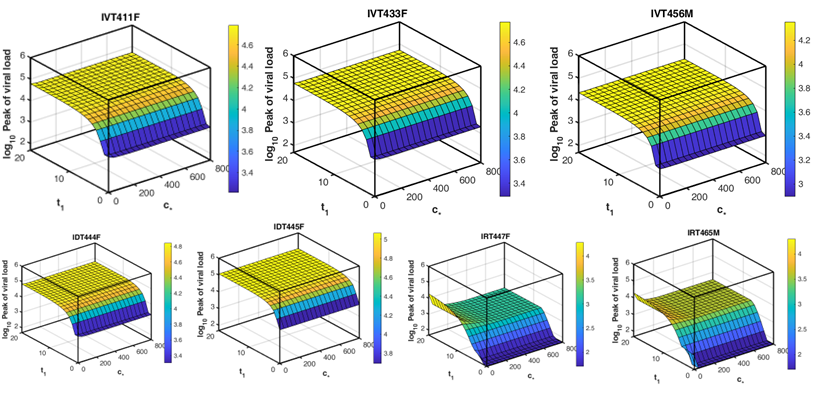

Supplement: S10 Fig — (TIF) [file pcbi.1013073.s010.tif]

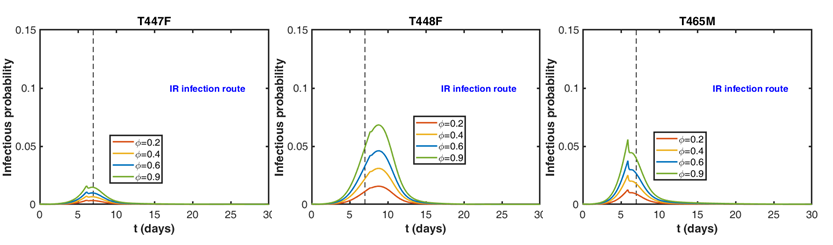

Supplement: S11 Fig — Different colors correspond to distinct ϕ values: orange (ϕ=0.2), blue (ϕ=0.4), green (ϕ=0.6), and red (ϕ=0.9). The vertical dashed line marks the time of symptom onset. (TIF) [file pcbi.1013073.s011.tif]
