# Supplementary material for: Uncovering the impact of infection routes on within-host MPXV dynamics: Insights from a mathematical modeling study
Source: PLoS Comput Biol. 2025 May 19;21(5):e1013073. doi: 10.1371/journal.pcbi.1013073 (PMC12088049; doi:10.1371/journal.pcbi.1013073)
Supplement: S1 Text — (DOCX) [file pcbi.1013073.s013.docx]

**S1 Text. Model data fitting and parameter estimation**

Data-fitting process

The data fitting process involves two steps. First, we fit four models to viral load data collected from 18 infected macaques by minimizing the least-squared residual error between observed data and the model predicted viral load on a logarithm scale. The optimization is performed using Matlab’s *fmincon* function (https://www.mathworks.com/help/optim/ug/fmincon.html), a widely used tool for solving constrained nonlinear optimization problems. Leveraging domain knowledge, biologically plausible parameter ranges are defined to ensure that the optimization process remains within realistic bounds. The *fmincon* function systematically searched the parameter space within these constraints to minimize the sum of squared residuals, achieving an optimal fit between model predictions and observed data.

The parameters estimated through data-fitting include the infection rate of cells by cell-free infection ($\beta$), the death rate of productively infected cells ($\delta$), the production rate of viruses ($p$), the effect of interferon in reducing infectivity of target cells ($\gamma$), the maximum effect of non-specific adaptive immune response ${(c}_{*}$), and the activated time of non-specific adaptive immune response ($\tau$). Since the *fmincon* function requires the specification of upper and lower bounds for each parameter, we selected biologically plausible yet sufficiently wide ranges to allow flexibility during optimization. For example, the death rate of productively infected cells ($\delta$) is expected to be higher than that of uninfected cells ($d_{1}=0.15$), thus the lower bound of $\delta$ was set to 0.2. The full set of bounds of estimated parameters used in the optimization is as follows: $\beta\in[0, {10}^{-3}], \delta\in[0.2, 1], p\in[100, 1500], \gamma\in[0, 1], c_{*}\in[200, 1200], \tau\in[5, 20].$ The code for generating results can be found at: <https://doi.org/10.24433/CO.4257228.v2>.

To further assess the robustness of our parameter fitting and examine whether the obtained solution corresponds to a global minimum, we applied a MultiStart global optimization approach. Specifically, we initiated the optimization from 15 random starting points within biologically plausible parameter bounds. All optimization runs successfully converged with positive solver exit flags, and the resulting fits exhibited highly consistent parameter estimates and viral load trajectories. These results support the conclusion that the fitting procedure did not become trapped in suboptimal local minima, and that the estimated parameters are robust to the choice of initial values. The MATLAB code used for the MultiStart optimization is included in this supplementary file.

Parameter estimation for the best model

After identifying the full model as the best model for capturing the viral load dynamics of 18 infected macaques in the first step, we proceeded to refine the parameter estimation using Bayesian inference. This was achieved by implementing the full model in *Stan* to fit the viral load data using a Bayesian framework.

The Bayesian approach integrates prior knowledge about the parameters with observed data to estimate posterior distributions. Specifically, we defined priors for key parameters of the model (e.g., infection rate, adaptive immune response activation time) based on domain knowledge, using distributions such as normal and exponential. The likelihood function was constructed using the logarithmic viral load data and modeled observation noise. Markov Chain Monte Carlo (MCMC) sampling via the No-U-Turn Sampler (NUTS) was performed to sample from the posterior distributions, with 4 chains and 2000 iterations per chain. Posterior samples were used to calculate the mean values of the parameters, as well as to generate model predictions. The predictions were obtained by solving the ODEs for each sampled parameter set, and 95% confidence intervals were constructed to capture prediction uncertainty.

The results indicate that the parameter means, and model predictions derived using Bayesian inference are very close to those obtained with the least-squares optimization method (*fmincon*). Both approaches successfully capture the observed viral load dynamics, but the Bayesian method provides additional insights by quantifying parameter uncertainty and prediction variability. This similarity confirms the robustness of the full model and the consistency of the fitting results across both estimation frameworks. The following figure is the posterior distributions for model parameters using observed data from macaque T409F. The posterior distributions of parameters for the remaining macaques should also be obtained. To save the space, here only present the results for one macaque. One can check result through the available code at: <https://doi.org/10.24433/CO.4257228.v2>.


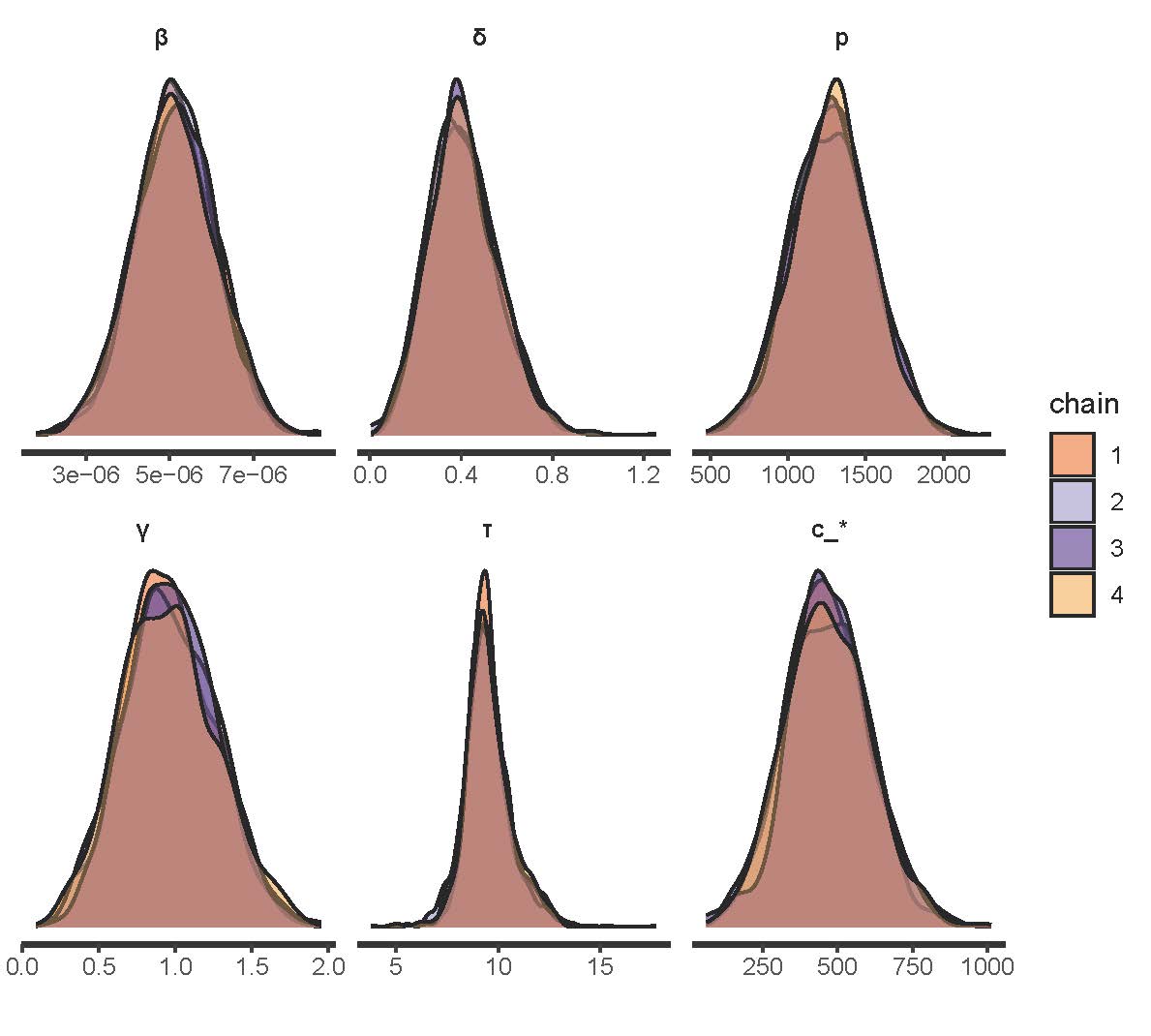


# *MATLAB Code for MultiStart Optimization (Example: Macaque #T409)*

%% viral load data from macaque #T409

ID_time = [0 3 7 10 14 21 28];

ID = [1.7 3.98 4.67 5.62 3.44 1.70 1.70];

%% Initial conditions

X0 = [2*10^5 0 0 50];

lb=[0 0.2 100 0 5 200]; %beta = par1(1); delta_0 = par1(2); pp = par1(3); gamma = par1(4); tau_1 = par1(5); c_max = par1(6)

ub=[10^(-3) 1 1500 1 20 1200];

par1guess=[10^(-6) 0.3 1200 0.08 9 450];

%% Optimization options

options = optimset('Display','off','MaxIter', 2000, 'MaxFunEvals', 2000);

%% Define objective function and optimization problem

objfun = @(par1) lsmethod(par1, ID, X0);

problem = createOptimProblem('fmincon',...

'objective', objfun,...

'x0', par1guess,...

'lb', lb,...

'ub', ub,...

'options', options);

%% Run MultiStart global optimization

ms = MultiStart('Display', 'iter', 'StartPointsToRun', 'all');

n_start_points = 15;

[par1, fval, exitflag, output, solutions] = run(ms, problem, n_start_points);

%% Optional: Check solution variability

all_solutions = reshape([solutions.X], length(par1), []);

std_sol = std(all_solutions, 0, 2);

disp('Standard deviation across solutions:');

disp(std_sol);

%% ===== ls and model =====

function d = lsmethod(par1, ID, X0)

try

[~, x] = ode45(@full_immune, 0:1:30, X0, [], par1);

xsimulation = [x(1,4), x(3,4), x(7,4), x(10,4), x(14,4), x(21,4), x(28,4)];

d = norm(log10(xsimulation) - ID')^2;

catch

d = Inf;

end

end

function dydt = full_immune(t, y, par1)

lambda_1 = 3e4; d_1 = 0.15; k = 4; c0 = 10;

beta = par1(1); delta_0 = par1(2); pp = par1(3);

gamma = par1(4); tau_1 = par1(5); c_max = par1(6);

c = c_t(t, c0, tau_1, c_max);

dydt = [

lambda_1 - d_1*y(1) - (beta/(1 + gamma*y(3)))*y(1)*y(4);

(beta/(1 + gamma*y(3)))*y(1)*y(4) - k*y(2);

k*y(2) - delta_0*y(3);

pp*y(3) - c*y(4)

];

end

function c = c_t(t, c0, tau_1, c_max)

if t < tau_1

c = c0;

else

c = c0 + c_max / (1 + exp(-(t - 1.7 * tau_1)));

end

end
